# Early Adipogenesis and Upregulation of UCP1 in Mesenchymal Stromal Cells Stimulated by Devitalized Microfragmented Fat (MiFAT)

**DOI:** 10.1155/2024/1318186

**Published:** 2024-09-11

**Authors:** Valentina Coccè, Sara Missaglia, Eleonora Martegani, Daniela Tavian, Luisa Doneda, Barbara Manfredi, Giulio Alessandri, Costantino Corradini, Aldo Giannì, Emilio Ciusani, Francesca Paino, Augusto Pessina

**Affiliations:** ^1^ CRC StaMeTec Department of Biomedical Surgical and Dental Sciences University of Milan 20122, Milan, Italy; ^2^ Laboratory of Cellular Biochemistry and Molecular Biology CRIBENS Università Cattolica del Sacro Cuore, Milan, Italy; ^3^ Department of Psychology Università Cattolica del Sacro Cuore, Milan, Italy; ^4^ Department of Biomedical Surgical and Dental Sciences Sports Trauma Researches Center University of Milan c/o 1st Division of Orthopedics and Traumatology Orthopedic Center Pini CTO-ASST Gaetano Pini, Milan, Italy; ^5^ Maxillo-Facial and Dental Unit Fondazione Ca' Granda IRCCS Ospedale Maggiore Policlinico 20122, Milan, Italy; ^6^ Department of Diagnostics and Technology Fondazione IRCCS Istituto Neurologico “C.Besta”, Milano, Italy

**Keywords:** adipogenesis, microfragmented fat, MSCs, UCP1

## Abstract

Adipose tissue is mainly composed by adipocytes. Moreover, mesenchymal stromal/stem cells (MSCs), macrophages, endothelial cells, and extracellular matrix components are present. The variety of molecules as cytokines and growth factors of its structure very rich in blood vessel makes it also similar to a true endocrine organ that however needs still to be fully investigated. In our study, we used human lipoaspirate to obtain mechanically microfragmented fat (MiFAT) which was washed and then devitalized by freezing–thawing cycles. In our experiments, thawed MiFAT was used to stimulate cultures of MSCs from two different sources (adipose tissue and gingiva papilla) in comparison with a traditional stimulation in vitro obtained by culturing MSCs with adipogenic medium. MSCs stimulated with MiFAT showed a very early production of lipid droplets, after only 3 days, that correlated with an increased expression of adipokines. Furthermore, a significant upregulation of PPAR gamma 1 alpha coactivator (PPARGC1A) was observed with an overexpression of uncoupling protein 1 (UCP1) that suggest a pattern of differentiation compatible with the beige–brown fat.

## 1. Introduction

Adipose tissue is an abundant tissue consisting of adipocytes, mesenchymal stromal/stem cells (MSCs), macrophages, endothelial cells, and extracellular matrix components. This type of tissue is easy to obtain by minimal invasive methods and also represents an important source of MSCs that can be isolated and expanded in vitro. Furthermore, MSCs differentiate in adipocytes, osteocytes, and chondrocytes and possess important regenerative capacity as confirmed by studies in animal models and also applied in human clinics [1–3].

The anti-inflammatory and cell protective properties of the fat tissue raised great interest. This has been due at first to the MSC secretome which contains specific cytokines, growth factors, and adipokines [4–6]. Furthermore, by considering that this tissue is very rich in blood vessel and contains such high variety of molecules, it assumes also an interesting importance as endocrine organ [7, 8]. The endocrinology function of adipose tissue remains an exciting research area not fully investigated, and many findings are still expected in particular concerning its possible role in the complex cross-talk between organs regulating the energetic functions including the insulin role, the lipid metabolism, and, probably, the immune system. The investigation on the secretion products of fat could be of interest both for new drug development and improved knowledges on the endocrinology of adipose tissue. This could improve the understanding of obesity, diabetes, and also cardiovascular diseases. As reported by Sarkanen et al. [9], cell-free extract from mature human adipose tissue was able to induce angiogenesis and adipogenesis in vitro by inducing triglyceride accumulation in human adipose stem cells. In our study, we investigated the ability of frozen microfragmented fat (MiFAT) to stimulate MSCs coming both from adipose tissue and from gingiva which have a different tissue of origin but also express a specific phenotype as previously described [10]. Our results evidenced a very early production of lipid droplets into the MSCs that correlated with an increased levels of adipokine expression and also a significant upregulation of uncoupling protein 1 (UCP1) protein suggesting a differentiation pattern compatible with beige–brown fat.

## 2. Materials and Methods

### 2.1. MSCs

As previously described, MSCs were isolated from adipose tissue lipoaspirates (AT-MSCs) or from gingival papilla tissue (GinPa-MSCs) of healthy donors according to standardized methods [10, 11]. Primary cultures were characterized for their proliferation rate, expression of the typical MSC markers [12], and multidifferentiative ability toward mesodermal lineage [13].

### 2.2. MiFAT

MiFAT specimens were obtained from lipoaspirate samples of three healthy donors, using a commercial device called “Lipogems®” as previously described [14]. Samples of subcutaneous adipose tissue were collected after signed informed consent of no objection for the use for research of surgical tissues (otherwise destined for destruction) in accordance with the Declaration of Helsinki. The approval for their use was obtained from the Institutional Ethical Committee of Milan University (no. 59/15, C.E.UNIMI, 09.1115). Aliquots of MiFAT were stored in a 15-mL tube at −20°C until use.

### 2.3. MiFAT Ultrafiltration

An aliquot of 5 mL of MiFAT was mixed with 1 mL of PBS and vortexed for 3 min and then the emulsion sonicated with 2 cycles of 40 s at a frequency of 0.4 pulses/second (Labsonic Braun apparatus). The sonicated MiFAT was placed in Centricon with 100 kDa membrane and centrifuged at 2500 × *g* for 20 min to obtain a sample of ultrafiltered MiFAT (MiFAT < 100 kDa) and an amount of unfiltered sample that did not pass through the filter (MiFAT > 100 kDa).

### 2.4. MSCs/MiFAT Cocultures

MSCs were seeded at a density of 2 × 10^4^ cells/cm^2^ in 25 cm^2^ flask in 4 mL of DMEM low glucose medium (Euroclone, Milan, Italy) supplemented with 5% platelet lysate Stemulate (Cook Regentec, Indianapolis, IN) for AT-MSCs, or 10% fetal bovine serum (FBS, Euroclone, Milan, Italy) for GinPa-MSCs, and 2 mM L-glutamine (Euroclone). After 24 h of incubation at 37°C, 5% CO_2_, 1 mL of MiFAT was added to the flask. Cellular monolayers were observed after 24, 48, and 72 h, until 4 days of incubation. Not treated cells were used as negative control. After 4 days of exposure, MiFAT was removed from flasks and cellular monolayer was washed three times with Hanks solution (Euroclone, Milan, Italy). To evaluate cellular proliferation, cells were stained with 0.25% crystal violet (Sigma-Aldrich, St. Louis, MO) for 10 min, washed with PBS buffer, and eluted with 0.3 mL of 33% glacial acetic acid. The absorbance of the eluted dye was measured at 550 nm. MSC population doubling time (PDT) after MiFAT exposure was calculated as follows: *DT* = Δ*t* × ln2/ln(Nc/Np), where Np is the number of plated cells, Nc is the number of counted cells, and Δ*t* is the time in culture expressed in hours.

### 2.5. Adipogenic Differentiation

To induce adipocytic differentiation, cells were plated in 35 mm^3^ petri dishes (Nunc, Germany) at density of 400 cells/cm^2^ in 1 mL/petri of their medium, which was replaced after 24 h with specific differentiation media supplemented with 500 *μ*M isobutyl-methylxanthine (AppliChem, Germany), 200 *μ*M indomethacin (Alexis Biomedical, United States), 1 *μ*M dexamethasone, 1 *μ*M hydrocortisone, and 10 *μ*g/mL insulin (all from Sigma-Aldrich, United States). After 15 days, cells were stained with Oil Red O solution (Sigma-Aldrich, United States).

### 2.6. Oil Red O Staining

The presence of lipid droplets was evidenced by Oil Red O Staining (Sigma-Aldrich, United States). After removing the supernatant from the culture plates, cells were washed once with phosphate-buffered saline (PBS) and fixed with 4% formaldehyde in PBS for 15 min at room temperature. The formalin was removed, and Oil Red O working solution was filled into the culture plates to safely cover the plate bottom (1.25 mL per well for 6-well plates). The plates were incubated at room temperature for 30 min. They were then washed five times with distilled water. To elute the dye, 100% 2-propanol was added to the plates (2.5 mL per well for 6-well plates). The plates were incubated for 10 min at room temperature. About 2× 200 mL of eluate was transferred to a clear 96-well microtiter plate (polystyrene); a duplicate of wells on the microtiter plate was filled with 2× 200 mL 2-propanol. Absorption was measured in triplicate at 545 nm by a Chromate microplate reading (Awareness Technology, Inc.).

### 2.7. Mitochondrial Labeling

The activity of mitochondria in both the AT-MSCs and GinPa-MSCs has been evaluated by MitoTracker™ Dyes for Mitochondria Labeling (Code M7512, Invitrogen, United States). Both AT-MSCs and GinPa-MSCs were cultured for 3 days in the presence of culture medium (CTRL) or adipogenic medium or MiFAT. Then, half of the cells from each treatment were incubated with 100 mM MitoProbe according to the procedure indicated by the company protocol, and the other half was harvested for autofluorescence control. All the cells were analyzed by flow cytometry (Navios EX, Beckman Coulter), and at least 25,000 events were considered using a specific software (Navios, Beckman Coulter). The increase of mean fluorescence intensity (MFI) induces by MitoTracker in cells was normalized as ratio on autofluorescence of plain cells.

### 2.8. RT-PCR Analysis and Primers

#### 2.8.1. RNA Extraction and Real-Time Quantitative PCR (RT-qPCR)

Total RNA was extracted from pellet of AT-MSCs or GinPa-MSCs alone or after 4 days of MiFAT exposure, following instructions of PureLink™ RNA Mini Kit (Thermo Fisher Scientific). RNA was treated with DNase to exclude DNA contamination, and then, 1 *μ*g of RNA was reverse transcribed into cDNA using QuantiTect Reverse Transcription Kit (Qiagen). Samples were analyzed using RT-qPCR. PCR reactions were performed using Rotor-Gene Q cycler (Qiagen), and the amplifications were carried out using the QuantiNova™ SYBR® Green PCR (Qiagen) in a total volume of 20 *μ*L. Real-time PCR was performed using the following primer sequences: ADIPONECTIN forward 5′-CAACATTCCTGGGCTGTACT-3′ and reverse 5′-CCTGTGAAGGTGGAGTCATT-3′; PPARG forward 5′-ACAGCAAACCCCTATTCCATGC-3′ and reverse 5′-ATTACGGAGAGATCCACGGAGC-3′; LEPTIN forward 5′-AAGCTTCAGGCTACTCCACA-3′ and reverse 5′–TGGAAGAGTGGCTTAGAGGA-3′; DGAT1 forward 5′-GCTTCAGCAACTACCGTGGCAT-3′ and reverse 5′-CCTTCAGGAACAGAGAAACCACC-3′; GPAT3 forward 5′-CCCAGAAGGGAGGCATTTGT-3′ and reverse 5′-GAACCTGGCCAACCATAGCA-3′; PPARGC1A forward 5′-GTTCCCGATCACCATATTCCA-3′ and reverse 5′-GCGGTGTCTGTAGTGGCTTGA-3′; UCP1 forward 5′–AGTTCCTCACCGCAGGGAAAGA-3′ and reverse 5′-GTAGCGAGGTTTTGATTCCGTGG-3′; RPLP0 forward 5′-TGGTCATCCAGCAGGTGTTCGA-3′ and reverse 5′-ACAGACACTGGCAACATTGCGG-3′. Data were analyzed by using the 2^−*ΔΔ*Ct^ method to obtain the relative expression level, and each sample was normalized by using RPLP0 RNA expression. The experiments were carried out in duplicate, and each data reports the mean value.

### 2.9. Western Blot

Cellular extracts were obtained from HeLa (positive control) [15], fibroblasts (negative control), or AT-MSC pellet using a buffer containing 1% NP-40 and freshly added protease inhibitors. The protein levels were determined using Bradford Protein Assay Kit (Bio-Rad). Proteins (25 *μ*g/well) were separated on a 10% SDS-polyacrylamide gel (Bio-Rad) and then transferred to a polyvinylidene difluoride (PVDF) membrane (Bio-Rad). After blocking with 5% nonfat dry milk solution, PVDF was incubated using a rabbit polyclonal antibody against UCP1 (dilution 1:500, GeneTex) and a mouse monoclonal antibody against *β*-actin (1:5000, Millipore). Detection was achieved with the Clarity Western ECL Substrate (Bio-Rad), and densitometric analysis was performed using Image Studio Digits, Version 4.0 (LiCor). The intensity of the bands of interest was normalized to *β*-actin.

### 2.10. Statistical Analysis

Data are expressed as average ± standard deviation (SD). Differences between mean values were evaluated by analysis of variance (ANOVA) using a Student–Newman–Keuls multiple comparison test performed by GraphPad Instat program (GraphPad Software Inc., San Diego, CA, United States). *p* values ≤ 0.05 were considered statistically significant. The linearity of response and the correlation were studied using regression analysis, by Excel 2013 software (Microsoft, Inc.).

## 3. Results

### 3.1. Effect of MiFAT on the MSC Growth

**Figure 1:**
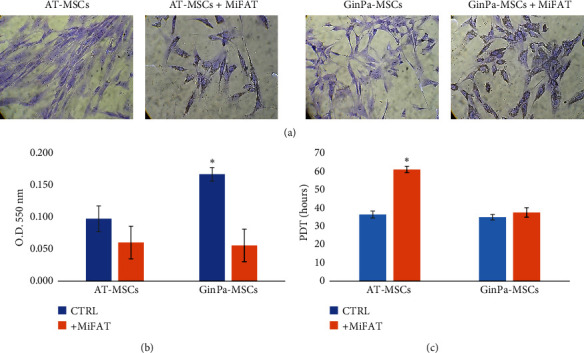
Effect of microfragmented fat (MiFAT) on mesenchymal stromal cells growth. (a) Representative cell images after crystal violet staining of adipose-derived mesenchymal stromal cells (AT-MSCs) and gingival-derived stromal cells (GinPa-MSCs), after 7 days of incubation, alone or in presence of MiFAT. Scale bar, 100 *μ*m. (b) Optical density (O.D.) at 550 nm after crystal violet elution with acetic acid. (c) Population doubling time (PDT) after 7 days of MiFAT exposure. Values are expressed as mean ± standard deviation (SD) of three replicates. ^∗^*p* < 0.05.

As evidenced by staining with crystal violet, both the adipose-derived mesenchymal stromal cells (AT-MSCs) and gingival derived stromal cells (GinPa-MSCs) cultured in the presence of MiFAT show some changes of their morphology. They lost the typical spindle-shaped morphology growing in parallel webbing orientation (Figure 1(a)) and showing the presence of a remarkable granulation of the cytoplasm. By evaluating the optical density (O.D.) at 550 nm after crystal violet elution with acetic acid, it was clearly evidenced that the presence of MiFAT in the medium reduced significantly (*p* < 0.05) the number of GinPa-MSCs in comparison with the controls (Figure 1(b)). Furthermore, while the PDT of GinPa-MSCs was not affected, the PDT of AT-MSC was almost two times increased (Figure 1(c)).

### 3.2. Effect of MiFAT on the MSC Adipogenesis

**Figure 2:**
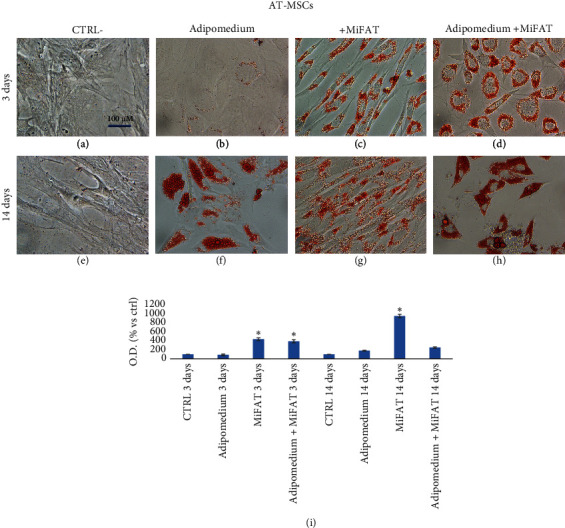
Effect of MiFAT on AT-MSC adipogenesis. The adipogenic differentiation was evaluated on AT-MSCs after 3 or 14 days of culture in (b, f) adipogenic differentiation medium or in presence of (c, g) MiFAT or (d, h) adipogenic medium + MiFAT by Oil Red O staining for lipid droplets. ((a, e) undifferentiated cells), 400× magnification. (i) The histogram represents the optical density (O.D.) at 545 nm after Oil Red O elution with 2-propanol (mean ± SD of three replicates). ^∗^*p* < 0.05.

**Figure 3:**
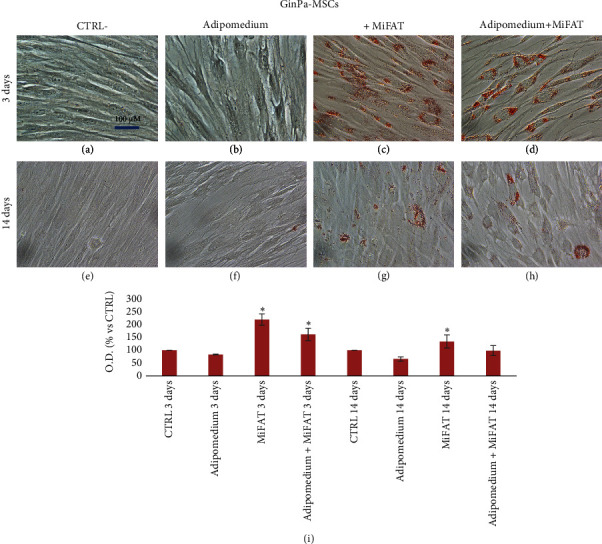
Effect of MiFAT on GinPa-MSC adipogenesis. The adipogenic differentiation was evaluated on GinPa-MSCs after 3 or 14 days of culture in (b, f) adipogenic differentiation medium or in (c, g) presence of MiFAT or (d, h) adipogenic medium + MiFAT by Oil Red O staining for lipid droplets. ((a, e) undifferentiated cells), 400× magnification. (i) The histogram represents the optical density (O.D.) at 545 nm after Oil Red O elution with 2-propanol (mean ± SD of three replicates). ^∗^*p* < 0.05.

**Figure 4:**
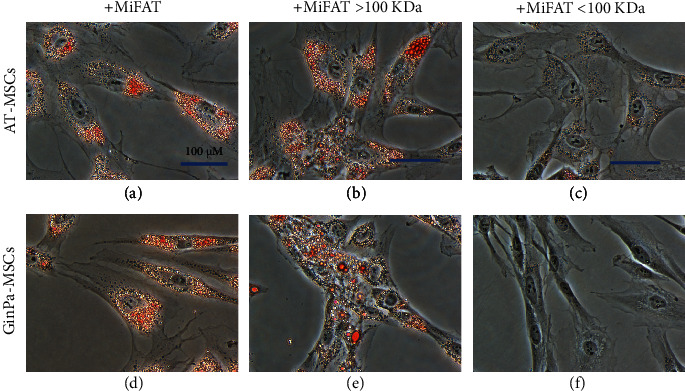
Effect of MiFAT on MSC adipogenesis. The adipogenic differentiation was evaluated on AT-MSCs or GinPa-MSCs after 3 days of (a, d) MiFAT or (b, e) MiFAT > 100 kDa and (c, f) < 100 kDa exposure by Oil Red O staining for lipid droplets. 400× magnification.

As expected, after 3 days of treatment of MSCs with a specific adipogenic medium, a very poor amount of lipid droplets stained with Oil Red O was observed in AT-MSC and no evidence in GinPa-MSCs (Figures 2(b) and 3(b)). However, if the two mesenchymal cells were stimulated with MiFAT alone or added to the adipomedium, the presence of numerous Oil Red O positive fat cells in both MSCs appears already after 3 days. (Figures 2(c), 2(d), 3(c), and 3(d)). At 14 days, a significant adipogenesis in AT-MSCs was observed while no adipocytes appeared to be present in GinPa-MSCs (Figures 2(f) and 3(f)). Whereas this strong adipogenesis pattern is maintained well expressed at 14 days in AT-MSCs (Figures 2(g) and 2(h)), a significant decrease of cell stained was observed in GinPa-MSCs (Figures 3(g) and 3(h)). This pattern of differentiation, also evaluated by the Oil Red O elution (Figures 2(i) and 3(i)), confirmed the previously reported differentiation character of GinPa-MSCs [10]. Furthermore, by studying the early adipogenesis at 3 days, it was observed that this stimulation to differentiate is obtained only by using the fraction of sonicates MiFAT greater than 100 kDa (> 100 kDa) (Figures 4(b) and 4(e)) while the treatment with the lower sonicated fraction (< 100 kDa) (Figures 4(c) and 4(f)) was completely ineffective on the two types of MSCs.

### 3.3. Expression of Adipogenic Markers by RT-PCR Analysis

**Figure 5:**
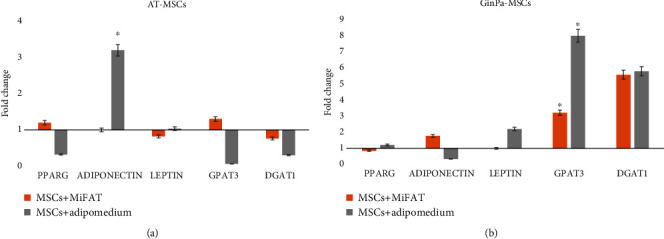
mRNA expression of adipogenic markers. Modulation of PPARG, ADIPONECTIN, LEPTIN, GPAT3, and DGAT1 in (a) AT-MSCs and (b) GinPa-MSCs after 3 days of exposure to MiFAT. Data are expressed as mean ± standard deviation (SD) of three replicates. ^∗^*p* < 0.05.

To evaluate the early adipogenic steps induced by MiFAT in comparison to the adipogenic medium of common use, we analyzed by RT-qPCR the expression of five genes.  Figure 5 shows that already after only 72 h of treatment, a significant modulation of gene expression was triggered both depending on the different treatment and on the type of MSCs. In particular, adipomedium treatment dramatically overexpressed adiponectin in AT-MSCs and downregulated the gene in GinPa-MSCs. On the contrary, MiFAT did not modulate adiponectin in AT-MSCs but enhanced its expression in GinPa-MSCs. The most interesting observation was the opposite behavior of PPARG, GPAT3, and DGAT1 genes in response to MiFAT evidenced in the two mesenchymal cell lines. Among these, relevant was the response of GinPa-MSCs in which both MiFAT and adipomedium induced a remarkable overexpression of GPAT3 and DGAT1 genes.

**Figure 6:**
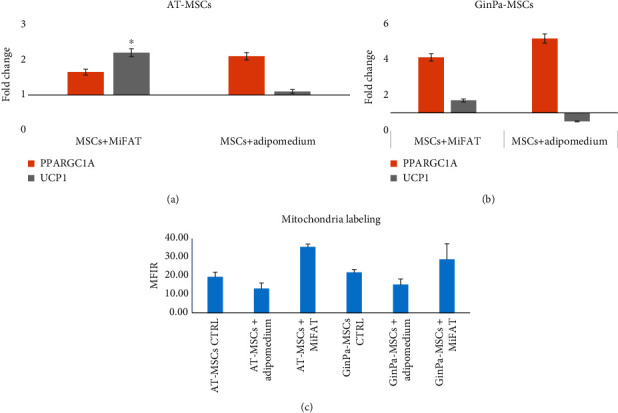
mRNA expression of PPARGC1A and UCP1 in (a) A-MSCs and (b) GinPa-MSCs after 3 days of exposure to MiFAT. At this time of treatment, also the mitochondrial labeling was evaluated as MFIR (mean fluorescence intensity ratio) and reported in histogram of (c). Data report the mean ± SD of a triplicate. ^∗^*p* < 0.05.

The results regarding the specific expression of UCP1 and PPARGC1A (Figure 6) demonstrated the notable overexpression of PPARGC1A and UCP1 induced by MiFAT in AT-MSCs and, in a lesser extent, also in GinPa-MSCs. Adipomedium did not modulate the UCP1 expression in AT-MSCs and produced a downregulation in GinPa-MSCs.

### 3.4. UCP1 Protein Expression

**Figure 7:**
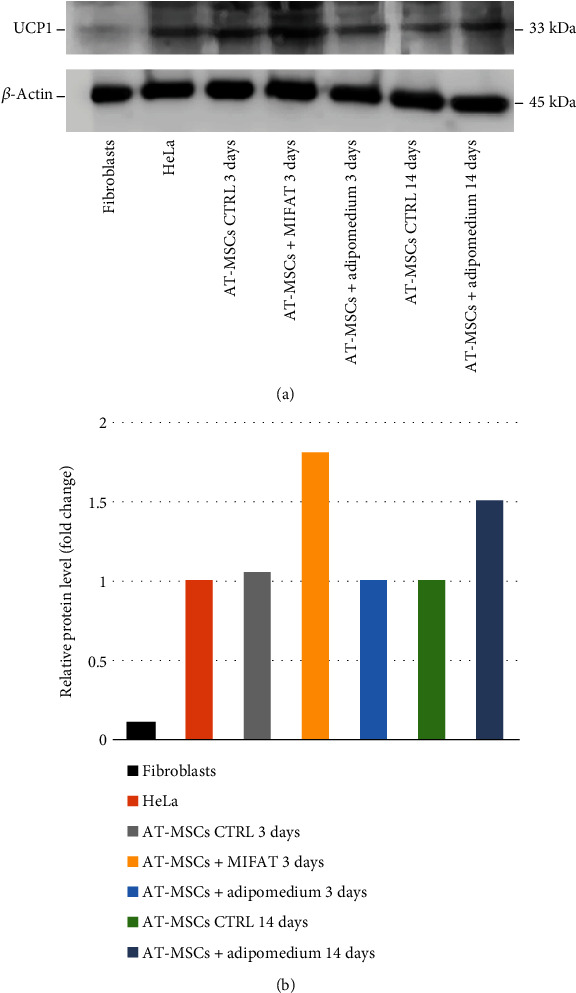
Western blot analysis of UCP1 protein expression. (a) Fibroblasts (negative CTRL), HeLa cells (positive CTRL), unstimulated AT-MSCs (CRTL at 3 days and 14 days of culture), and AT-MSCs stimulated 3 days and 14 days with MiFAT or adipomedium. (b) Densitometric analysis of Western blot.

The expression of UCP1 protein was evaluated by Western blotting analysis (Figure 7). The study showed that in MSCs, this protein is basally expressed as shown in CTRL after 3 days of culture in amount near to the level of HeLa cells used as positive control. A significant increase of protein expression was observed at 3 days when MSCs were cultured in the presence of MiFAT and after 14 days if cultured in adipomedium. GinPa-MSCs exhibited a very low level of protein expression that was not modulated by MiFAT and adipomedium treatment (data not shown).

### 3.5. Mitochondrial Labeling

The activity of mitochondria was evaluated by flow cytometry and expressed as MFI ratios. No significant increase in mitochondrial labeling was observed in AT-MSCs and GinPa-MSCs after 3 days of treatment with adipogenic medium. In both cell types, mitochondrial labeling was significantly higher (*p* < 0.05) when cells were stimulated by MiFAT suggesting an important activation that is generally recognized in the differentiation toward beige/brown adipocytes (BAs) (Figure 6(b)).

## 4. Discussion

In the preliminary experiments, a significant different effect exerted by devitalized MiFAT on MSC proliferation was evidenced. Whereas the duplication times of MSCs isolated from gingival papilla did not appear to be affected by MiFAT, on the contrary, MSCs isolated from adipose tissue significantly slow down the growth. This suggests that MiFAT secretome could exert different effect on cell cycle of MSCs if they are isolated from different sources (Figure 1). In effect, we cannot exclude some toxic effect of MiFAT on cell population or some interference with the adhesion capacity of cells. Furthermore, a role can be exerted by the push of the differentiation process being known that cellular maturation can significantly reduce their proliferative capacity to the point of eliminating it.

We know that, independently of their source, MSCs can differentiate into several lineages and, in the presence of specific factors, they differentiate into mature adipocyte [13, 16]. In our study, we confirmed this ability when MSCs were cultured for 14 days, but surprisingly, we found that, after only 72 h of treatment with MiFAT, the MSCs became positive for OilRed O staining by evidencing lipid droplets (Figure 2). The decrease in adipocytes evidenced in GinPa-MSC could be the result of balance proliferation/differentiation and also related to some described process of ECM remodeling during adipogenesis as indicated by the transformation of spindle-shaped preadipocytes into spherical adipocytes [17] This observation was also confirmed by stimulating MSCs with MiFAT sonicated and fractioned by ultrafiltration at 100 kDa (Figure 2). Only fraction > 100 kDa maintained the capacity to induce OilRed O positive cells in 72 h, by indicating that the induction of adipocytes is probably not due to some molecule present in the MiFAT with MW minor than 100 kDa. This agrees with our previous study [5] evidencing that MiFAT releases a combination of cytokines affecting several mechanisms involved in inflammation processes, but these molecules do not seem relevant to explain our results of early adipogenesis observed. As MiFAT is rich in lipid microdroplets having size > 100 kDa, we evaluate if our finding was only a physiological not specific consequence of some ability of MSCs to incorporate microdroplets.

By investigating the expression of five main adipokines involved in the MSC adipogenesis (Figure 5), the RT-PCR analysis evidenced, already at 72 h of treatment with MiFAT, a significant early upregulation of genes demonstrating that, in some way, an adipogenesis process was induced by MiFAT secretoma with a biogenesis of lipid droplets. In fact, the early commitment process from MSCs to preadipocytes is not completely known; adiponectin is considered a very early gene upregulated [18] and has been reported that PPAR*γ* in some condition were specifically upregulated later in the differentiation [19].

An apparent discrepancy emerges when comparing the trend of differentiation in adipocytes at 3 days versus 14 days (Figures 2 and 3) referred to GinPa-MSCs. In effect, this result on GinPa-MCS remains difficult to explain, but it completely agrees and confirms the behavior of these cells which, as previous reported, in the presence of adipogenic stimuli for 14 days produced few cells with lipid droplets [10]. On the contrary, of great interest in our present study, is the high number of GinPa-MSCs differentiated at only 72 h of stimulation with MiFAT.

Between the two MSCs, significant differences in the modulation of these genes were observed depending of the responses of the cell strains, and of interest is the significant upregulation of DGAT1 and GPAT3 genes that codify two enzymes localized in the mitochondrial outer membrane and endoplasmic reticulum (ER), respectively [20]. Their expression was more evidenced in GinPa-MSCs that are MSCs derived from gingiva. As known, GPAT3 gene is highly expressed in many human tissue and in 3T3-L1 cells where has been identified as a critical regulator for lipid accumulation during adipocyte differentiation [21, 22]. DGAT1 has revealed an important role in triglyceride synthesis for its ability in averting ER stress and lipotoxicity when lipolysis is stimulated in adipocytes.

However, if all the above discussed data demonstrated the well-known ability of MSCs to differentiate into white adipocytes [16], we also investigated their ability to differentiate also into BAs following some path like that observed by De Gemmis et al. [23] and regarding subcutaneous white adipocytes that expressed this capacity. To clarify this aspect, we studied both the expression of the mitochondrial UCP1 and PPR gamma 1 alpha coactivator (PPRG1A) that are considered important markers of brown adipose tissue, and we found (Figure 6) that these genes were not only basally expressed both by AT-MSCs and GinPa-MSCs but they were significantly overexpressed by MiFAT stimulation. As reported by many authors [24–26], in general, we know that PPARs are key mediators of lipolysis-induced activation of genes involved in lipid metabolism and thermogenesis and that PPAR*γ* is a nuclear receptor highly expressed in BAT that works as a master transcriptional regulator of BA differentiation. Furthermore, the transcriptional coactivator PGC-1*α* (797 aa), encoded by the PPARGC1A gene, is a key regulator of cold-induced BAT thermogenesis.

The more remarkable result was that, in the presence of MiFAT, a very early synthesis of UCP1 was triggered after only 3 days, and this agrees also with the significant increase of the mitochondrial activity (Figure 6(c)). Of course, these data do not prove that mature functional BAs are present in our cultured MSCs. However, the increased mitochondrial activity indicates that, in this differentiation step, the enhanced mitochondrial activity is compatible with their thermogenic and metabolic function related to the UCP1 mediated proton leak in BA [27, 28].

Also highlighted by western blotting alone, it is important to note that MiFAT modulates UCP1 mRNA translation in AT-MSCs (Figure 7). In fact, the MiFAT treatment of MSCs produced a very early increase (3 days) of this already expressed protein while such a level of expression was obtained only after 14 days of treatment with adipomedium. Furthermore, this behavior was not observed in GinPa-MSCs in which the increased mRNA expression of UCP1 after their treatment did not correspond to an increased protein production. Together, these observations agree with the well-accepted point of view that mRNA of UCP1 is not exclusive to brown adipose tissue, and it is basally expressed in many cell types, and its translation can be also easily modulated [29].

Although the steps regulating UCP1 transcription are rather complex and not fully investigated, the UCP1 gene has been identified an enhancer region with binding sites specific for different transcription factors. Some studies reported that in vivo UCP1 expression is induced by stimulation of the sympathetic system during cold exposure mediated by activation of *β*-adrenergic receptors. This leads to cAMP production and activation of cAMP-dependent protein kinase (PKA) and p38 mitogen-activated protein kinase (p38 MAPK) that in turn regulates the transcription of nuclear factors such as peroxisome proliferator-activated receptor gamma (PPAR*γ*) and the PPAR*γ* coactivator 1a (PGC1a) that have a pivotal important role in regulating transcription of UCP genes [30–33]. As described by Echtay et al. [34], in brown tissue, the long-term regulation of UCP1 depends on MAPK that leads to gene transcription and protein synthesis whereas in the acute regulation, the same MAPK can activate lipases to hydrolyze triglycerides by producing fatty acids that act as direct activator of UCP1. We know that microfragmented adipose tissue contains fatty acids and is also rich of triglycerides; in our model, it is possible that MiFAT triglycerides dispersed in culture medium are easily subjected to hydrolysis phenomena that produced a further increase of concentration of fatty acids that activate UCP1. In conclusion, our results suggest that MSCs have basal expression of UCP1 gene and that its mRNA expression and translation can be stimulated by MiFAT probably due to the presence of significant amount of triglycerides and fatty acids.

## Data Availability

All relevant data can be found within the article.
